# Immune Reconstitution Inflammatory Syndrome Secondary to Autoimmune Hemolytic Anemia and Cryptococcal Meningitis

**DOI:** 10.3389/fneur.2019.00812

**Published:** 2019-08-06

**Authors:** SiJia Yin, Yan Xu, Jinsha Huang, Nian Xiong, Chao Han, Kai Ma, Xing Fang Guo, Yun Xia, Fang Wan, Liang Kou, Yadi Sun, Jiawei Wu, Tao Wang

**Affiliations:** ^1^Department of Neurology, Union Hospital, Tongji Medical College, Huazhong University of Science and Technology, Wuhan, China; ^2^Department of Life Sciences and Medicine, The First Affiliated Hospital, University of Science and Technology of China, Hefei, China

**Keywords:** cryptococcal meningitis, autoimmune hemolytic anemia, leukoencephalopathy, immune reconstitution inflammatory syndrome, Cryptococcal Meningitis-Immune Reconstitution Inflammatory Syndrome

## Abstract

A 47-year-old HIV-seronegative woman with autoimmune hemolytic anemia (AIHA) was treated with corticosteroids for 8 months. She developed central nervous system dysfunction and was diagnosed with cryptococcal meningitis (CM) after detecting cryptococcus neoformans in the cerebrospinal fluid. The patient's clinical symptoms were worsened and unusual MRI findings of white matter lesions were noticed even after adequate treatment, which were quite unusual compared with typical characteristics of CM. This led us to carry out further investigations. Similar cases have been reported previously in published literature. Combined with clinical symptoms and MRI findings, the most likely diagnosis was Cryptococcal Meningitis-Immune Reconstitution Inflammatory Syndrome. Unfortunately, the patient deteriorated and died of respiratory failure. Cryptococcal Meningitis-Immune Reconstitution Inflammatory Syndrome may have MRI changes during the early onset of the disease (bilateral basal ganglia). We propose that close monitoring of the condition, meticulous MRI follow-up and brain biopsies should be indicated in such cases for treating them actively, so as to avoid worsening of the patients' condition.

## Background

According to literature reports, it is rare for patients with cryptococcal ceningitis (CM)/autoimmune hemolytic anemia (AIHA) to develop leukoencephalopathy. For CM patients without HIV infection, radiographic images frequently show no abnormality; less commonly, hydrocephalus orgadolinium enhancement of meninges may be seen. Immune Reconstitution Inflammatory Syndrome (IRIS) is a type of leukoencephalopathy, the appearance on neuroimaging of contrast enhancement, interstitial edema, mass effect, and restricted diffusion in infections are not typically characterized by these findings but raise strong suspicion for CM-IRIS. CM-IRIS may be more common in HIV+ patients, but for HIV- patient with AHIA and CM, this is the first report that takes into consideration the diagnosis of CM-IRIS.

## Case Presentation

A 47-year-old woman presented to the emergency department with a 2 months history of intermittent headache and a 6-day history of left hemiplegia. She had been diagnosed with AIHA 8 months earlier and required immunosuppressive treatment with corticosteroids to maintain a hemoglobin level >65 g/L, when the patient was presented with anemia, edema, rash, and fever as main clinical features. At that time, she also suffered from eosinophilia and hypoproteinemia. Lung CT scans indicated a pulmonary infection. Positron emission tomography (PET) imaging showed lymphadenitis and excluded lymphoma preliminarily.

**Figure 1:**
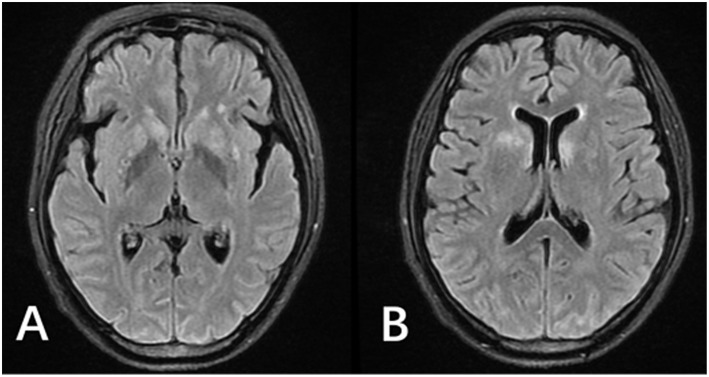
MRI of the patient after antifungal medication for 2 weeks. (**A,B)** FLAIR images showed hyperintense signals in the bilateral lateral ventricle white matter and bilateral basal ganglia. Enhanced T1 showed leptomeningeal enhancement in parts of the cerebral hemisphere and cerebellar hemisphere (not shown).

Two months before admission, increasing edema, rash, and fever developed again. Blood tests revealed eosinophil count 13.79 × 10^9^/L (total white cell count 24.11 × 10^9^/L, lymphocyte count 4.12 × 10^9^/L). Blood parasites identification was negative and no organisms were cultured from it. Hypersensitive C-reactive protein levels fluctuated. Serology was negative for both syphilis and HIV. His medications were prednisone (20 mg/day, then tapered), albumin infusion, imatinib, antibiotic, and antiallergic drugs. During the treatment period, the patient complained of a headache for the first time, which was accompanied with an intermittent fever. Moreover, she developed visual problems, such as diplopia and reduced acuity. Aside from a stiff neck, her physical examination was unremarkable. Lumbar puncture revealed cerebrospinal fluid (CSF) cell count 347 × 10^6^/L, white cell count 147 × 10^6^/L, glucose 4.47 mmol/L, protein 1.96 g/L, CSF IgM <6.94 mg/L, IgG 126 mg/L; the cryptococcus neoformans was detected in the CSF. Funduscopic examination shows binocular papilledema. Brain MRI showed increased enhancement of leptomeninges, fluid-attenuated inversion recovery (FLAIR)/T2 hyperintense in sulcus. The patient was given amphotericin B (AMB 40 mg/day) and voriconazole (0.2 g/day) treatment. Her headache remitted after 6 days. Repeat lumbar puncture after 2 weeks showed cell count 82 × 10^6^/L, white cell count 82 × 10^6^/L, glucose 3.16 mmol/L, protein 0.54 g/L, and again, cryptococcus neoformans was detected. Repeated brain MRI scans revealed new abnormal signal in the white matter of the bilateral ventricles and the basal ganglia areas (Figures 1A, B, **3A,B**). However, no further examination was performed due to improved symptoms. She returned to the local hospital to continue treatment.

**Figure 2:**
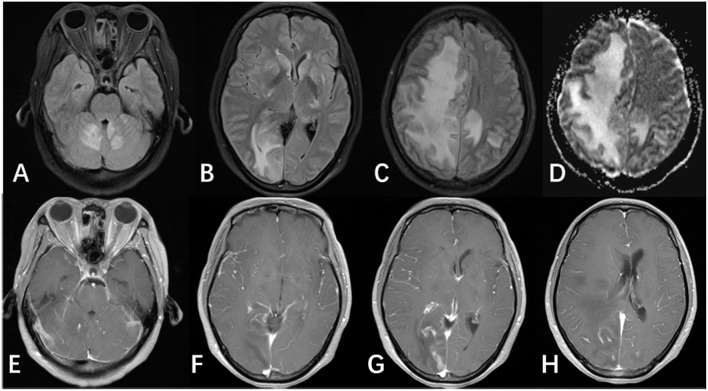
MRI of the patient after antifungal medication for 8 weeks. **(A–C)** FLAIR image showed multifocal lesions involving the cerebellar vermis, cerebellar hemispheres, bilateral basal ganglia, and the bilateral cerebral hemisphere. Images also showed large areas of edema and midline shift. **(D)** ADC and DWI images (not shown) showed hyperintense signals, and did not show evidence of restricted diffusion. **(E–H)** Enhanced T1 showed patchy enhancement in the bilateral basal ganglia, the cerebellar vermis, and the cerebellar hemisphere, in addition to leptomeningeal enhancement in the bilateral cerebral hemispheres.

**Figure 3:**
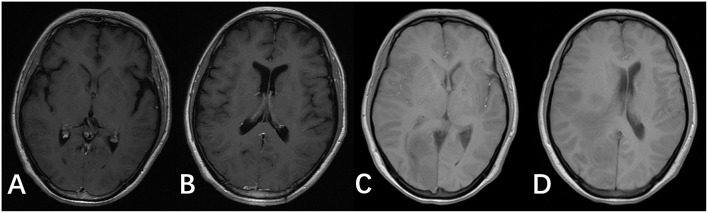
T1 images of the brain after antifungal medication for 2 and 8 weeks. (**A,B)** T1 images of the patient after antifungal medication for 2 weeks. (**C,D)** T1 images of the patient after antifungal medication for 8 weeks.

After 7 weeks of antifungal treatment, on February 23rd, 2018, the patient was referred to our department complaining of an increasing headache and a 6-day history of left hemiplegia. Clinical manifestations include intermittent low-grade fever, pneumonitis, altered mental status, and worsening visual problems. CSF examinations revealed cell count 297 × 10^6^/L, white cell count 197 × 10^6^/L, glucose 3.84 mmol/L, protein 1.13 g/L, CSF IgM 12.5 mg/L, and IgG 726 mg/L. No organisms (include cryptococcus neoformans) were detected in the CSF. Blood and sputum cultures were negative for any organisms. Treatments were switched from AMB and voriconazole to AMB and flucytosine. Repeated brain MRIs revealed significant lesions: a large, confluent area of FLAIR hyperintensity in the bilateral basal ganglia, and the white matter of the bilateral cerebral hemispheres, vermis of cerebellar, as well as the cerebellar hemisphere (Figures 2A–C, 3C,D). There was no evidence of restricted diffusion; however, there were signs of edema in the right hemisphere in addition to a midline shift of 0.9 cm. Enhanced T1 showed contrast enhancement in the bilateral basal ganglia, cerebellar vermis, cerebellar hemispheres (Figures 2D–H), not to mention bilateral leptomeningeal enhancement of the cerebral hemispheres. MRI imaging, obtained 1 month earlier, was significantly different from this presentation, but yet the PCR for CSF JCV DNA was negative (<2,000 copies/ml). Two weeks later, the patient's condition became even worse; a fever emerged, and she complained of lethargy and showed signs of altered mental status; 0.5 g/day × 3 methylprednisolone and symptomatic treatment slightly improved her symptoms. Unfortunately, she showed progressive neurologic deterioration and died of respiratory failure.

## Discussion

We presented a case of a 47-year-old HIV-seronegative woman with AIHA and eosinophilia, who received corticosteroid therapy over 8 months. We found that the patient did meet the atypical symptoms of Drug Rash with Eosinophilia and Systemic Symptoms (DRESS) before the diagnosis of AIHA was made. Her symptoms included a rash, eosinophilia, fever, facial edema, and a lung infection. After diagnosis of AIHA, we used corticosteroids for AIHA treatment. The rash gradually subsided and eosinophil levels were controlled. Looking back at the high-risk drugs that the patients were exposed to 2–6 weeks ago, we considered the possibility of β-lactam antibiotics acting as a trigger for DRESS syndrome. According to previous data, AIHA is also likely to be the sequela of DRESS syndrome. In this immune status, the patient developed symptoms of headache and was diagnosed as CM after cryptococcus neoformans was detected in the CSF. However, after a period of adequate treatment, the patient was presented with new and worsening clinical symptoms (hemiplegia, fever, pneumonitis, altered mental status, and worsening visual problems). Brain MRI showed a large, confluent area of FLAIR hyperintensity (1, 2), bilaterally in the basal ganglia, in addition to the white matter of the bilateral cerebral hemispheres, the vermis of the cerebellar, as well as the cerebellar hemispheres. All these symptoms are manifested as a type of leukoencephalopathy (3).

Why the patient presented with worsening clinical symptoms and unusual MRI findings is a question that interests us. In Table 1, we illustrate partial case reports of leukoencephalopathy secondary to AIHA or CM. Reviewing the literature, possible diagnoses include CM-IRIS, the toxicity of Amphotericin B, CM itself, acute disseminated encephalomyelitis (ADEM), and progressive multifocal leukoencephalopathy (PML). Based on the patient's clinical presentation and MRI findings, we consider the most likely diagnosis to be CM-IRIS.

**Table 1:** Case report of leukoencephalopathy secondary to AIHA or CM.

**Reference**	**Age/gender**	**Co-morbidities**	**Diagnosis and diagnosis basis**	**White matter damaged area**	**Treatment**	**Outcomes**
Sato et al. (4)	39/M	CM	L-AMB-induced leukoencephalopathy	Bilateral frontal and left occipital lobes	Discontinuation of L-AMB	Disappearance of almost all white matter lesions
Liu et al. (3)	43/M	CM	AMB-induced leukoencephalopathy: received amphotericin B i.v. at a total dose of 91 mg over 5 days; Brain biopsy	Multifocal white matter lesions	Low-dosesteroid	Clinical condition was partially reversed
Hajra et al. (5)	15/F	AIHA	ADEM: clinical manifestation and MRI	Multiple subcortical lesions of varying size	steroid	Responded well to steroid therapy. No residual lesion.
Jaing et al. (6)	16/F	AIHA CM	ADEM: clinical findings and MRI	Extensive white matter of the basal ganglia and bilateral frontoparietal lobes.	IVIG	Remarkably improved in clinical and MRI.
Wilcox et al. (7)	18/M 51/M	CM hypogammag lobulinemia CM chronic sinusitis	CM itself: Clinical symptoms, white matter changes were prior to AMB, Brain biopsy	Patchy subcortical Extensive right frontoparietal	AMB Fluconazole Monthly IVIG AMB	Responded well
Weitzman et al. (8)	–	Lymphoma CM	PML: discordant clinical and laboratory responses to CM treatment, as well as CT scanning imaging	(CT scanning) proved helpful in pointing toward PML	–	–
Kuwahara et al. (9)	72/M	Sweet disease CM	CM-IRIS: clinical manifestation, MRI and pathological findings	Cerebral deep white matter	Methylprednisolone AMB	Patient died ofrespiratory failure

In this kind of patient, a broad differential diagnosis is necessary. A small number of cases reported that AMB may cause leukoencephalopathy. Typically, cerebral MRI lesions of AMB-associated leukoencephalopathy showed hyperintensity on T2-weighted and FLAIR images. Furthermore, the lesions appeared predominantly in the frontal areas without gadolinium enhancement. Only after 2 weeks of treatment with antifungal drugs (AMB), an MRI of the patient showed bilateral lateral ventricular white matter and bilateral basal ganglia lesions. After 7 weeks of antifungal treatment, the MRI lesions of our case were mainly in the occipital lobe and extended to broad areas. The possibility that the toxicity of AMB causing white matter lesions can be ruled out. Patients with CM may be established with leukoencephalopathy and usually appear before or at the same time as CM diagnosis. However, in our patient, leukoencephalopathy did not appear at the same time as CM. Although new abnormal signals (white matter of the bilateral ventricle and the basal ganglia areas) (10) occurred 2 weeks after adequate treatment, in our case, leukoencephalopathy was less likely to be caused by CM. Only two articles suggested that there may have a relationship between leukoencephalopathy ADEM and AIHA. ADEM as a complication of AIHA has never been proven in the relevant literature. Furthermore, the patient's MR imaging progress was taken more than 1 month, while the occurrence of ADEM was more urgent. So, the diagnosis of ADEM is not supported. PML can be excluded based on imaging results. On MRI, the lesions of PML are seen as areas of decreased signal on T1images and increased signal on T2/FLAIR. PML lesions are usually bilateral and localized preferentially in the periventricular areas and the subcortical white matter, and generally do not enhance with contrast or to develop surrounding edema. Also, a negative result (JCV DNA <2,000 copies/ml) was tested, which can help to rule out the diagnosis of PML.

Retrospectively in her medical history, the earliest abnormality beyond CM imaging appeared after 2 weeks of adequate treatment. Her brain MRI scan revealed abnormal signal in the white matter (11) of the bilateral ventricles and basal ganglia areas. After 7 weeks of antifungal treatment, repeated brain MRIs showed severe lesions. Besides MRI imaging, in line with the imaging features of CM-IRIS, our patient suffered from a suspicious DRESS and had a history of autoimmune disease, which may cause a decrease in CD4+ T cell counts, followed by an increase in CD4+ T cells after corticosteroids and antifungal therapy. As well as microbiological clearance being documented by negative CSF cultures. These potential changes possibly developed her excited yet dysregulated inflammatory response to dead or latent organisms, which notably is the characteristic of IRIS. As brain biopsy remains the gold standard for the diagnosis of IRIS, further confirmation requires evidence of brain biopsy.

IRIS is a condition that occurs in immunosuppression or AIDS cases. The immune system recovers after treatment, but then responds to previously acquired opportunistic infections with an overwhelming inflammatory response that paradoxically makes the symptoms of infection worse (12). The mechanism of IRIS is thought to be due to a switch from following initiation therapy from a predominantly Th2 immune response (anti-inflammatory) to a Th1 or proinflammatory response. This robust inflammatory response can vary from mild to fulminating. IRIS is difficult to diagnosis as its clinical manifestations are not specific and may be caused by a variety of pathogens: HIV, opportunistic infection, unknown antigens, and so on. According to the criteria (13, 14), CNS-IRIS is a diagnosis of exclusion. Cases of IRIS need to have an inflammatory component that occur in the setting of immune reconstitution, which cannot be explained by drug toxicity or a new opportunistic infection. In general, IRIS can be recognized by atypical worsening clinical symptoms after adequate therapy, differences on MRI or CT, and pathological reports that indicate the pathophysiology of lymphocytic inflammation. In a CM-IRIS infected patients' brain, an MRI typically reveals leptomeningeal enhancement, which can be accompanied by a communicating hydrocephalus. Linear perivascular enhancement in the sulci and new meningeal or choroid plexus enhancement are also shown to be imaging indicators of CM-IRIS (15). Distention of the Virchow-Robin space manifests as a high T2/FLAIR signal, particularly in the basal ganglia; however, enhancement of these Virchow-Robin spaces appears to be characteristic of CM-IRIS, as does secondary involvement of the brain parenchyma characterized by areas of high T2/FLAIR signals. Lesions that develop surrounding edema during effective therapy are typically due to an immunologic response associated with IRIS. Kuwahara et al. (9) reported a case of CM-IRIS, characterized by abnormal intensities predominantly in the deep white matter. Perivascular infiltration of lymphocytes was evident in the deep cerebral white matter (16). Given that the majority of HIV+ patients have a lower CD4+ T-lymphocyte count prior to starting highly active antiroviral therapy (HAART) and respond to HAART in terms of rising CD4+ T cell counts (17) and falling HIV-1 RNA levels are associated with IRIS.

When early signs of IRIS occur, meticulous clinical and MRI follow-ups are indicated. Glucocorticoids may be useful as there are no controlled trials examining the use of glucocorticoids for IRIS; the dose and duration of treatment are unclear. We typically initiate 0.5 to 1 mg/kg of prednisone daily (or equivalent) and then taper the dose over the next 2 to 6 weeks. However, we do not routinely administer glucocorticoids at the start of cryptococcal meningoencephalitis treatment.

We summarized the possible causes of her condition, in addition to the diagnostic process, she underwent, and the corresponding solutions of leukoencephalopathy secondary to AIHA and CM (see Flow chart 1). A summary of this case can yield the following conclusions: for patients with autoimmune disease combined with CM, meticulous clinical and MRI follow-up in combination with brain biopsy is warranted. Furthermore, lymphocyte clustering can aid diagnosis when we suspect IRIS. If we closely monitor the clinical condition and MRI changes of the patient, we can actively treat the patient as soon as we observe lesions to the patient's basal ganglia. This can potentially save the patient's life.

## Data Availability

All datasets generated for this study are included in the manuscript and/or the supplementary files.

## Ethics Statement

Written informed consent has been obtained from the patient's husband for the publication of this case report.

## Consent for Publication

Written informed consent has been obtained from the patient's husband for publication of this case report and any accompanying images. A copy of the written consent is available for review by the Editor-in-Chief of this journal.

## Author Contributions

SY and YXu collected and analyzed data and wrote the manuscript. TW revised the manuscript for intellectual content. JH, NX, CH, KM, and XG critiqued the content, and proofread the manuscript. YXi, FW, LK, YS, and JW contributed to the manuscript content, and approved the final version.

### Conflict of Interest Statement

The authors declare that the research was conducted in the absence of any commercial or financial relationships that could be construed as a potential conflict of interest.
